# Web-Based Co-design in Health Care: Considerations for Renewed Participation

**DOI:** 10.2196/36765

**Published:** 2023-03-03

**Authors:** Maryam Mallakin, Christina Dery, Samuel Vaillancourt, Sahil Gupta, Katherine Sellen

**Affiliations:** 1 Health Design Studio OCAD University Toronto, ON Canada; 2 Department of Medicine University of Toronto Toronto, ON Canada; 3 Department of Emergency Medicine St Michael’s Hospital Toronto, ON Canada

**Keywords:** web-based design research, co-design, web-based co-design, virtual platform, virtual learning platforms, internet research ethics, collaboration, health communication, sensemaking, health design, tangible tools and games

## Abstract

The COVID-19 pandemic has shifted the work environment to a new reality of remote work and virtual collaboration. This shift has occurred in various work settings with an impact on spaces, approaches, applied techniques, and tools. This has resulted in the broad use of virtual tools in the health care sector to avoid physical encounters and in-person interactions that will likely outlast the COVID-19 pandemic. Developing effective virtual approaches requires the knowledge and skills of using digital technologies collaboratively combined with a deep understanding of the context or contexts in which these approaches may be used. The implementation of virtual health design methods, including web-based co-design, has increased to meet the realities of COVID-19 restrictions and is likely to outlast them. Adapting the use of co-design methodologies to a virtual configuration requires rethinking methods of collaboration and communication, adapting to virtual environments, and creating new methods of engagement and facilitation. With this viewpoint, we reviewed the current work on co-design (in person and web based) to propose techniques for the design, planning, and implementation of web-based co-design. We propose 7 considerations that may enable web-based co-design projects in the health care sector. The key considerations that affect the success of a web-based co-design approach should be considered in the process of planning, developing, and conducting web-based co-design sessions. These include facilitation, collaboration, accessibility and equity, communication, sensemaking, tangible tools and games, and web-based research ethics. We illustrate this work with a case study of co-design for an emergency department discharge tool developed during the pandemic.

## Introduction

### Background

In recent years, co-design methods have been widely applied in health care systems [1]. The application of these methods is rapidly expanding, specifically in the new era of remote work and virtual collaboration owing to the COVID-19 pandemic.

A co-design approach provides an opportunity to share, mobilize, and activate knowledge by engaging patients and health stakeholders in a collaborative research and design process [2]. Co-design is a design-led approach to change, with a set of creative and participatory principles, practices, and tools. Co-design has the potential to be used in many areas of health care such as improving the quality of care and patient experience [3] by drawing on a collaborative and equitable lens that brings health stakeholders and patients together to explore complex problems. Various activities and tools (eg, tangible tools, design games, and play-like activities) are used in co-design processes to support idea generation and foster communication among participants. These in-person activities and tools highlight the exploratory, imaginative, dialogical, and empathic aspects of co-design [4]. Ordinarily, these tools are used directly by participants in person and have not been actively developed for virtual spaces or modes before the COVID-19 pandemic.

The Covid-19 pandemic has resulted in a dramatic shift in the use of digital technologies, the internet, and internet-based services for communication, interaction, and collaboration in all aspects of work and life [5]. Many people now have increased exposure to web-based engagement and collaboration as well as a greater willingness and the required skills to engage in web-based activities [6]. As a result, virtual connections have become more acceptable, and digital engagement opportunities have increased and diversified [6]. The pandemic required many groups to begin working remotely, including design researchers and practitioners who needed to shift to web-based design activities [6]. Although the COVID-19 pandemic has increased the development and application of web-based co-design methods and tools, they are not limited to the pandemic and can be used as an extension to co-design practices even after the pandemic.

Collaborative activities and tools for both in-person and web-based co-design are central to facilitating meaningful and productive engagement and collaborative discussions among stakeholders. The use of tangible tools, game-like activities, and scenarios can elicit novel responses on a subject matter because the playfulness of these activities tends to foster creative behavior [7]. Imaginative and pretend play are effective strategies for idea generation and for moving toward mutual or shared understanding. Brown and Vaughan [8] emphasize the importance of “play” or “tinkering” for problem-solving and working with our hands (tangible experiences) to “see solutions” that otherwise would not be seen [7], an aspect largely missing in web-based experiences. In addition, communication is a powerful tool in any game-like activity and plays an essential role in participatory activities such as co-design techniques [8,9]. Although in-person and web-based communication share common characteristics, web-based communication has additional challenges that should be addressed, such as the lack of nonverbal cues that can compromise connection and empathy [10]. Lack of tangible interaction and compromises to connection are just 2 of the several issues that must be addressed in web-based co-design [11]. Although there are challenges, web-based co-design may also afford a broader reach for stakeholder engagement, with the constraints of a physical location and day or time meeting removed as barriers for some.

### Objectives

The objective of this viewpoint is to describe a set of 7 considerations that were developed in response to the pandemic, in the design, development, and conduct of web-based co-design, and applied in the health sector.

## Methods

In this viewpoint, we have captured considerations for web-based co-design in health care emerging from adaptations in co-design practices undertaken at the Health Design Studio, OCAD University, Canada during the pandemic. These considerations represent a combination of continuous review of the literature for possible adaptation strategies and the experience of adapting a project to test these considerations during the pandemic (illustrated by the case study below). In our review, we aimed to identify from existing and prepandemic studies the main challenges and opportunities for participatory design approaches and co-design activities in a web-based setting, specifically focused on web-based co-design within the health care system. We used an exploratory approach for this literature review, combining insights from research articles with available guidance and resources and other gray literature [12]. In parallel, we explored and made adaptations in an ongoing project (case study), adapting processes and techniques to achieve web-based co-design for a co-design project originally situated within an emergency department (ED). The case study is used in this viewpoint to illustrate adaptations of co-design during a pandemic in context [11,13].

The case study (Designing Discharge after Emergency Care [D.DEC] project [14]) was a parallel activity to experiment with techniques and draw additional insights about the main challenges, opportunities, benefits, and drawbacks of web-based co-design for health. The D.DEC project aimed to develop an improved and appropriate patient-centered approach for discharge information in the ED. As the COVID-19 pandemic emerged, the project had to move its co-design work to web over the duration of the project. As the team working on the D.DEC project, we were in a position to have firsthand experience with the adaptations made to the project and how those adaptations affected the project. We undertook the literature search as we developed adaptations, took notes as we went, and wrote this viewpoint together to capture the knowledge created through this experience. Ethics approval for the D.DEC project was granted by both the design research team’s institution and the hospital in which the ED was situated.

## Results

### Overview

The web-based search identified information from the academic literature as well as relevant toolkits, handbooks, reports, guidelines, webinars, and presentations in the gray literature. Search terms included “co-design,” “codesign,” “participatory,” “participatory design,” “participatory design tools,” “participatory approach in health,” “health communication,” “co-design in health,” “web-based communication,” “virtual learning,” “sensemaking,” “internet research ethics,” “synchronous & asynchronous communication tools,” “design games,” “virtual play and creativity,” and “health, health care,” “health sector,” “medical,” and “web-based design research,” “virtual collaboration platforms,” “web-based or remote co-design,” “guides on digital accessibility,” “web-based tools for design thinking,” and “internet research ethics.”

Two researchers reviewed the abstracts of candidate articles for relevance to the aim of the literature review. Three researchers read each article for a final set of 52 peer-reviewed articles for inclusion in the review. The gray literature, in which design-related practice is more often documented, included a search for co-design toolkits (n=2), co-design handbooks (n=2), collaboration challenge (in-person or web-based) reports (n=4), facilitating virtual meeting guidelines (n=3), and webinar guidance (n=2). We retrieved resources from 15 organizations and institution websites, specifically design organizations and design in health groups.

We used a virtual meeting platform (Zoom, Zoom Video Communications) and a web-based visual collaboration platform (Miro) to conduct remote collaborative teamwork during the research process. The Miro web-based whiteboard was used to house articles and resources and share and study our findings, enabling us to explore and experience some of the challenges and opportunities of a web-based collaborative setting.

### Case Study

The D.DEC project [15] was carried out in the ED in an urban center and focused on discharge information as a key opportunity to improve patient outcomes beyond the care provided in the ED. As stated above, the D.DEC project was one of the several projects undertaken by the Health Design Studio at Ontario College of Art and Design University when the pandemic hit. The project’s co-design approach was intended to bring diverse stakeholders together to identify a creative solution for developing a precise, feasible, sustainable, and patient-centered tool. Initially structured as an in-person project with 2 large multistakeholder workshops centering on the co-design process, the actual process consisted of the following eight steps: (1) review of existing research, (2) intensive observations, (3) collaborative synthesis, (4) co-design sessions 1a and b, (5) web-based feedback, (6) co-design session 2a and b, (7) prototype implementation with feedback and testing, and (8) refinement of the solution. The project became an opportunity to experiment with adaptations in the co-design process and techniques through web-based means.

Shifting to a web-based co-design mode was an opportunity for the project team to learn about the challenges, gaps, and potential opportunities in making that shift. In total, 25 stakeholders participated in co-design sessions 1a and b and 2a and b, including patients, emergency physicians, emergency nurses, and family physicians, with the aim of discussing, identifying, and developing an improved method of discharge information in the ED setting. The first round of web-based co-design sessions (1a and b) focused on the discharge information delivery process and identifying design needs, and the second round of co-design sessions (2a and b) reviewed and refined possible design solutions. To support the delivery of these virtual sessions, Zoom was used as a web-based meeting platform, Google slides was used for sharing screens and documents, and Google Docs was used to take meeting notes together. The project team identified two main challenges in conducting the web-based co-design sessions:

1. Technical challenges and capabilities related to using web-based platforms
2. The level of participation, collaboration, and interaction from participants, which led to mostly conversations around existing knowledge instead of generative ideation and creating new knowledge.

The design of a web-based co-design process, specifically addressing health-related interventions, requires strong knowledge of the context and skills in problem formulation, shared processes, problem-solving, and collaborative solutioning. An appropriate web-based system is required to support a practical collaborative space with a variety of participation opportunities and tools [16].

Conducting and facilitating a web-based co-design process consists of 3 main phases [17]: the preworkshop phase (planning process), the workshop phase (conducting process), and the postworkshop phase (data analysis and evaluation process).

Well-planned virtual processes rely on applying the right combination of web-based or offline and synchronous or asynchronous tools to enable opportunities for both facilitators and participants to feel empowered during the co-design process. Providing combinations and alternatives supports participants in managing their time, space, and feedback and enables facilitators to analyze the outcomes and adjust their methods and agenda throughout the process [18].

### Seven Considerations for Web-Based Co-design in Health

#### Overview

Through our scoping review and exploration of the D.DEC case study, we collated insights about various cocreation elements and the importance of selecting appropriate collaboration tools to improve participation, discussion, and ideation among participants. We identified 7 factors that affected participants’ engagement and collaboration in a web-based co-design process (Textbox 1).

**Factors affecting participants’ engagement and collaboration in a web-based co-design process.**

1. Facilitation
2. Collaboration
3. Accessibility and equity
4. Communication
5. Sensemaking
6. Tangible tools and games
7. Web-based research ethics

The following section includes brief descriptions of these 7 factors, how each of these factors is reflected in the D.DEC project, and recommendations based on insights from the academic and gray literature.

#### Facilitation

Facilitation plays a critical role in providing meaningful cocreative opportunities (structure and space) in co-design to guide participants through sessions and ultimately plays a central role in facilitating the uncovering of new data and insights from participants. Facilitation requires expertise, resources and preparation for planning engagement, prioritizing tools, and exploring creative solutions [19].

It is more challenging to build an effective, creative, and encouraging collaboration in a virtual setting, making facilitator roles even more important than in-person settings.

In the planning and preparation phase of the D.DEC web-based co-design sessions, we thoughtfully developed a facilitator script to ensure that the facilitators created a collaborative and welcoming space. The script addressed ethics, accessibility, digital literacy, tone, language, and the specific activities and processes of the co-design sessions. The creation of the script served both the training and evaluation roles. We conducted a test web-based co-design session with participants naive to the project to refine materials and scripts, develop facilitators’ comfort level with web-based delivery, communication channels, and facilitation of collaboration tools and materials. Two facilitators took turns to either facilitate dialogue and collaborative creation or manage technology, materials, and multiple communication channels.

Facilitators play a central role in web-based co-design to provide well-planned and focused processes, ensure equal opportunity for contribution from all participants, and keep participants motivated and focused [17]. Facilitation tasks in web-based co-design can be divided into 5 main categories: methodology, technical administration, content, user interaction, and results [17]. The facilitator’s role includes deciding the topic, setting the context, planning rules and the agenda, inviting participants, providing access and motivation, managing and adapting technical considerations, stimulating discussions, identifying new topics, recognizing contributions, maintaining participation, and wrapping up. Having a trained and prepared facilitation team (consisting of the main facilitator, cofacilitators, and technical assistant for technical administration and support) and a well-planned process play an essential role in conducting a successful web-based co-design process [17].

#### Collaboration

Collaborating and designing with participants is the main component and central activity of a design-based research process. Collaboration within a co-design process includes communication, cooperation, and cocreation [10]. The original D.DEC project was structured in a 2-tiered manner with a core collaborative team including designers and physician leads with 2 large multistakeholder workshops. The workshops were intended to serve multiple purposes, including socializing the project with decision makers and eliciting design considerations from a broad range of roles in the ED. This approach was chosen in part owing to restrictions on in-person involvement in activities for ED staff, because a large meeting sanctioned by department decision makers was one mechanism for enabling unionized and highly time-constrained staff involvement. The pandemic hit just before the first workshop of 35 participants.

Given the variability and constraints of availability among the stakeholder groups (patients, emergency physicians, nurses, clerks, and family physicians) during the pandemic, the team decided to provide opportunities for feedback from frontline staff using web-based surveys between smaller and more frequent co-design sessions. This was deliberately intended to maintain a broader collaboration by providing access to otherwise unavailable stakeholders. Facilitation in the co-design session included deliberate focusing and privileging of diverse voices (focusing on roles other than emergency physicians who were already central to the project). In switching to a web-based engagement strategy, one of the challenges identified in the D.DEC web-based workshop was participant capabilities related to the use of the selected platform, which affected their engagement and collaboration in discussions.

There are various factors that affect participants’ collaboration in a web-based co-design process in a health context such as an ED, including (1) selecting the right platform, programs, and tools that are appropriate for engaging participants in selected activities and tasks, specifically selecting tools that are equity positive by requiring as little technological proficiency as possible and the least sophisticated equipment as possible; (2) initiating initial interaction with participants to foster better relationships by establishing trust, connection, and commitment; (3) removing time constraints and planning shorter and more frequent sessions (eg, multiday engagement activities) to provide more flexible engagement for health care staff and patients, and providing facilitators the opportunity to analyze data and adjust agendas and activities to support continued contributions; (4) planning the right combination of synchronous or asynchronous activities and tasks to provide time and space for participants to manage their ideas; (5) including trained facilitators to avoid biased discussions and discussion breakdown, and providing better opportunity for participants to form shared understanding and commitment to the project’s goals; (6) applying techniques and activities that are interactive, understandable, pleasing, and engaging; and (7) dividing participants into smaller groups by topic and logistics (eg, who is technology savvy? Who is comfortable with the camera? Which health care roles experience power dynamics that might restrict their voice) [10].

#### Accessibility and Equity

Although web-based research methods are rapidly expanding (accelerated by the COVID-19 pandemic) and becoming commonplace in health research, there are various challenges that affect their effectiveness. Access to digital technology and equitable resources, including time and space to participate, can affect the ability to maintain participation across stakeholders.

The main challenges identified through the D.DEC web-based co-design process include the distribution and access to digital resources (eg, hardware, software, and internet), level of skills required for participation (eg, literacy level and familiarity with computer technology and programs), and privacy and security considerations (eg, availability of staff spaces, family, culture, anonymity, and confidentiality) [20]. For some patients, digital video was not an option, and a physical version of the materials was mailed to their home, and participation by phone was made available. Flexibility and facilitation were key to maintaining access to the session and participation at the same level as other participants.

Accessibility requirements for patients with disabilities or impairments should be included in the guidelines for selecting platforms and activities that can fully support various needs such as hearing, vision, or speech impairments. It is important to select platforms that are compatible with assistive technologies (eg, screen readers) and accessible for people who are deaf or hard of hearing, blind or visually impaired, have sensory disabilities, and have intellectual or developmental disabilities [21]. In each collaborative case, the accessibility features of the platforms should be evaluated to establish sufficiency of access for the specific case and its participants. Among the various available platforms, Zoom and Microsoft Teams are 2 platforms that provide more inclusive (although, not entirely) virtual accessibility features for collaborative activities [22]. Providing a paper version of materials, asynchronous participation, or offline participation option should also be considered, especially when vulnerable groups and issues of health care access are central to the co-design project.

#### Trauma-Informed Practices

Remembering or recounting negative or harmful experiences in health care spaces may be traumatizing for participants. Addressing trauma is an important component that should be considered to maximize safety, accessibility, and equity in a web-based co-design process. Trauma-informed care is a strengths-based approach “that is grounded in an understanding of and responsiveness to the impact of trauma, that emphasizes physical, psychological, and emotional safety for both providers and survivors, and that creates opportunities for survivors to rebuild a sense of control and empowerment” [23]. The integration of trauma-informed care principles is critical for fostering more accessible and safe spaces when hosting virtual meetings (eg, web-based co-design). The 6 key principles of a trauma-informed approach are as follows: emotional and physical safety; cultural, historical, and gender considerations; trustworthiness and transparency; peer support and mutual self-help; collaboration and mutuality; and empowerment, voice, and choice [24].

#### Communication

Communication is a powerful tool that plays an essential role in participatory techniques. Communication should be accessible, inclusive, and generate shared understanding and empathy among designers, researchers, and stakeholder groups [9].

The initial co-design activities for the D.DEC project included a large-scale workshop of 20 to 35 people across various roles within the ED, with the addition of patients and family physicians. Larger-scale co-design activities are effective mechanisms for expressing complex issues and building empathy and understanding across disparate health care stakeholders. However, in the context of web-based co-design, we chose to reduce the scale, opting for 1 to 2 stakeholder representatives per stakeholder group per session. In this manner, the affordances of the Zoom platform, equal visual representation and single person auditory focus, mute function, and hand raising, could be used to reduce existing power dynamics that would otherwise have affected communication balance. We also chose to use previously crafted patient stories (video based) to communicate the focus of the co-design session, centering around patient voices from the beginning.

Effective communication requires an in-depth understanding of the context, priorities, needs, beliefs, environment, social norms, and preferences of the intended audiences. Communication in co-design creates consensus and ownership of the process and its outcomes [9]. Virtual and in-person communication may share common principles and motivating factors to enhance participants’ engagement, but they require different implementation paths. The main communication challenges in virtual settings that may lead to confusion and misunderstanding include (1) lack of nonverbal cues such as body language, facial expression, and eye contact; (2) lack of strong connection, empathy, and trust among participants; and (3) lack of control over the process (for participants) [10].

To address these challenges, the study by McCarthy et al [12] suggests using multimedia platforms such as web-based meeting platforms (Zoom, Microsoft Team, etc) that support the use of nonverbal cues such as tone of voice, eye contact, or facial expression and dividing participants into smaller groups (5 people) to provide better opportunities for connection. Applying methods and tools such as storytelling, storyboards, and scenarios can help improve connections among participants to enhance emotional reciprocity, shared understanding, trust, and empathy [25].

#### Sensemaking

During in-person co-design, materials and interactions affect sensemaking. When co-design is conducted through web, the technical aspects of the experience play a role in the sensemaking process. Technical sensemaking refers to participants’ interactions with technology that could be challenging, such as how to use technology or how to handle technological failures (eg, video freezing or audio cutting out). Interpersonal sensemaking refers to participants’ interactions with other participants, which could be challenging owing to a lack of motivation or communication gaps (eg, lack of interpersonal feedback with those who are trying to communicate). There is a shortcoming in the literature about sensemaking for virtual environments that might otherwise point to adaptations that can be made to facilitate the move to web-based co-design. Story and narrative are strong sensemaking tools that play important roles in information sharing, collective interpretation of problems, and improving communication [26].

Supporting sensemaking for the D.DEC co-design sessions included carefully created visual support for each co-design subactivity and a highly structured co-design workshop agenda and script. This enabled open dialogue around specific aspects of the design process, shared sensemaking on gaps, and provided possible solutions for discrete aspects of the co-design of the discharge tool. We used filmed, re-enacted patient stories to try to bring about a sense of empathy. We also included multiple check-in points and verbal reiterations of insights and ideas, conclusions, and suggestions at each subactivity by the facilitator.

The existing literature largely focuses on addressing our understanding of sensemaking for in-person contexts [27]. Amber and Jorgen [27] point to unique aspects of the virtual environment that affect the sensemaking process, including (1) partial presence that may limit the capacity to detect opportunities for interaction and sensemaking; (2) concurrent states of being “in” and “out,” which means the participants can be in 2 places at once, (ie, at their PC in their home or office and in the virtual space), which can add a new dimension and complexity to sensemaking in virtual environment owing to moving “in” and “out” of the virtual world; (3) disembodiment, which means subconscious, physical cues that are normally used to communicate with one another and make sense are lost in virtual settings; and (4) no known etiquette or norms in virtual environmental interactions that refer to the set of rules and norms that are followed in real-world interaction, which are ambiguous or nonexistent in virtual settings.

#### Tangible Tools and Games

A participatory design approach typically includes a variety of techniques and tools to engage participants in collaborative discussion and co-design, depending on the topic of the research, types of participants, and circumstances under which the research is conducted [17]. Tools are the material components that are used to connect design and research practices in the participatory co-design process [28] and can include probes, tangible tools, and games.

Probes are participatory design tools that often consist of material objects (eg, disposable cameras, postcards, stickers, maps, and art materials). Probes are often exploratory in nature and are intended to enhance dialogue and invite participants to be involved in different phases of the exploratory design process, including (1) probing knowledge and meaning, (2) provoking reflections, (3) projecting visions or ideas into the future, and (4) prototyping ideas and concepts that envisage future reality [29].

Tangible tools include visual tools such as graphic representations and artifacts and generative tools such as scenario boards, storyboards, videos, and collages [28]. Tangible tools are intended to enable collaborative, innovative, and active dialogue among participants in the design process. Tangible tools are defined as materials used in participatory design activities to facilitate knowledge exchange; shared understanding; and generating ideas among participants through making, telling, and enacting approaches.

Design games are generative, visual, and playful tools used to transfer knowledge and ideas and generate new ideas and insights into the co-design process, often including shared decision-making mechanisms. Design games have various applications in collaborative and participatory processes, including supporting creative thinking, engaging and empowering participants in an exploratory and human-centered design process, enhancing social collaboration, and understanding individual participants’ experiences [30]. The exploratory design game framework by Brandt [31] takes advantage of the various skills and expertise of the participants to generate new ideas and design possibilities in the participatory process. The framework consists of various exploratory design games, including games to conceptualize designing, “exchange perspective,” design games, negotiation and workflow-oriented design games, and scenario-oriented design games [31].

During the D.DEC project, when considering the design of participatory engagement as the team moved toward prototyping discharge communication options, it became necessary to develop activities that would engage a range of stakeholders in exploring new ideas about communication techniques and the content of discharge communication. Given the diverse technology access and acceptance among participants, the team chose to create tangible materials to represent different potential design options and to physically send them to all participants. These paper-based materials included prompting steps and options for participants to contribute during the session both verbally and by making notes or using stickers to provide feedback on each design option.

Many tangible or probing tools can be used in a web-based co-design approach to engage participants in web-based collaboration and cocreation (telling, making, and enacting) [32]. The following tools can be used in a web-based co-design process:

- Visual tools: sketches, diagrams, visual and graphic representations, and video
- Generative tools:
  Telling: stories, storyboarding, self-observation (photo taking, short video, and drawing), diaries, voting, stickers, sorting, and categorizing to prioritize ideas
  Making: 2D collages and 2D mapping
  Prototyping apps (eg, Boards, Mockingbird, and Pop)
  Enacting: scenario making, participatory envisioning, and improvisation
- Virtual design games for shared decision-making and prioritization.

### Web-Based Research Ethics

Web-based research has uncovered new ethical challenges for researchers, requiring new considerations for various aspects of recruitment and participation. Despite the growing interest in web-based research, the ethical guidelines and policies needed to guide these practices are insufficient [33]. Web-based research refers to situations in which researchers set the research context as one with a significant interaction between the researcher and participants in a web-based setting.

Participants’ privacy (ie, family and cultural considerations), anonymity and confidentiality, informed consent, and data security and integrity are some of the ethical challenges that should be considered in web-based research settings. Factors related to these challenges include the audience (with whom to consult), type of research activity, and epistemological perspectives (space or place and text based or person based), informed consent (public-private, degree of interaction, topic sensitivity, and subject vulnerability), researcher ethos (credibility and variability of roles), and ethical representation (publication in the age of remix, multimedia, and search engines) [20]. It is important to consider the different ethical issues involved in various types of web-based research. “Each type of research involves different levels of involvement and interaction from both the participant and researcher. The more involvement and interaction, the greater, one can assume, the ethical risk may be” [33].

Because the D.DEC project included a diverse participant group with a range of digital literacy and access, it was important to create an equitable and inclusive experience for participation. We created options for participation both in how and when to participate, as well as asynchronous and synchronous options, including flexibility during a session. Asynchronous activities, such as asynchronous feedback, increase flexibility and extend the potential breadth of participation [34]. We provided phone call availability for answering questions and put in place multiple facilitators to help ensure equal voice when a variety of engagement techniques or technologies were in use (phone or chat or virtual meeting or paper based). In addition, the identification of participants (names and faces) and recording of sessions were 2 other ethical challenges in the D.DEC project.

## Discussion

### Principal Findings

Web-based co-design methods and techniques have become increasingly common across industries, settings, and professions [35]. Consequently, conducting remote and virtual co-design has become an opportunity to advance participatory techniques. This viewpoint presents considerations for using a co-design developed from inquiry and adaptations during the pandemic. We examined the recent challenges in conducting co-design and identified potential opportunities to address them for projects conducted for a health context. The web-based co-design phases from the D.DEC case study revealed some of the drawbacks and challenges of the web-based setting, including technical challenges and capabilities related to using web-based platforms and the level of participation, collaboration, and interaction among participants. Through a literature review and scanning of web-based or offline co-design resources, we identified the main factors that can affect co-design in virtual settings, including facilitation, collaboration, platform accessibility, communication, sensemaking, using tangible tools, and web-based research ethics. These factors are intended to improve communication, increase shared understanding, support effective sensemaking, and support meaningful discussion among participants, which in turn may improve interaction, collaboration, and the generation of new ideas and creative solutions.

In developing a set of considerations for web-based co-design in health, we looked at existing work, including the participatory framework proposed by Sanders et al [28]. Sanders et al [28] proposed that a design framework can help design researchers determine which participatory techniques and tools are most relevant for a specific design process. The framework by Sanders et al [28] provides an overview of participatory design tools and techniques in virtual and in-person settings that may be complementary to the 7 considerations shared in this viewpoint. The framework by Sanders et al [28] is intended to orient practitioners to the purpose and context of participatory tools and techniques and to support the customization of those tools and techniques [18]. Identifying the project’s context, purpose, and goals is an essential step in planning a participatory research process in both the real world and virtual settings [10]. Important considerations for planning include (1) the context of the project including the purpose, goals, and objectives; (2) the target participants, in terms of numbers, abilities, motives, background, and experiences; (3) the characteristics and agenda of the process (eg, outputs and communication characteristics); (4) the characteristics of activities and tasks (eg, types of activities in terms of form, complexity, and timing); (5) the platforms and tools that fit with the goals and outcomes of the project; and (6) web-based research ethics [10]. These 6 aspects of the framework focus on planning co-design, and we would recommend consulting the framework alongside the 7 more conceptual considerations for co-design more so than some of the practical aspects.

In support of co-design outcomes, tools and techniques should aim to improve idea generation by facilitating communication and interaction among participants throughout the process (eg, visual tools such as graphic representations and generative tools such as cards and storyboards). Tangible tools, design games, and play-like activities are used in co-design to highlight the exploratory, imaginative, dialogical, and empathic aspects of co-design in improving idea generation and fostering communication between participants. “The means for reaching these objectives are drawn up in addition to the design (eg, tangible mock-ups and user representations) from the world of games (eg, role-playing, turn-taking, make-believe) to deliberately trigger participants’ imagination as a source of ideation for problem solving” [6]. There are several ways through which “play” can be initiated into a virtual co-design setting such as “play triggers” involving physical, verbal, or situational factors [7]. “Play” can be supported through tangible materials (such as game boards, playing cards, or prompt cards) and rules to provide a starting point or signal to the overall tone and expectations of a free and safe space to explore imaginative thoughts and ideas in a low-fidelity manner.

From a more practical perspective, it is critical to select the right platform or combination of software and platforms, activities, and materials that support the inclusivity of diverse participants in the research process. Well-planned web-based methods such as co-design workshops rely on integrating alternative access and communication methods to enhance inclusivity through increased accessibility. Alternatives include sharing information and materials via mail. Conducting and facilitating a web-based co-design process consists of 3 main phases [17]: the preworkshop phase (planning process), the workshop phase (conducting process), and the postworkshop phase (data analysis and evaluation process). Well-planned virtual processes rely on applying the right combination of web-based or offline and synchronous or asynchronous tools to enable opportunities for both facilitators and participants to feel empowered during the co-design process. Providing combinations and alternatives supports participants in managing their time, space, and feedback and enables facilitators to analyze the outcomes and adjust their methods and agenda throughout the process [18]. In addition, to address the challenge of agency over the process, applying a combination of web-based or offline and synchronous or asynchronous tools and techniques can help integrate opportunities for both facilitators and participants to include agency in the co-design process. This offers participants flexibility in managing their time, space, and feedback and enables facilitators to analyze the outcomes and adjust their methods and agenda as they see fit [18].

### Conclusions

In recent years, participatory methods, including co-design, have been integrated into health care. The application of these methods in a web-based setting is rapidly expanding, specifically in the new era of remote work and collaboration owing to the COVID-19 pandemic. Adapting and using participatory design methods in a web-based setting requires the knowledge and skills to combine offline and virtual technologies, virtual collaboration, and creative methods and techniques.

We have been able to integrate existing work on the practical and conceptual aspects of co-design together with practical experience by adapting a co-design project for web-based engagement in the health sector. We present 1 example, but there are many projects that have experimented with adaptations out of necessity during the pandemic. Further research is required to fully capture the learnings from these experiences to improve co-design and to effectively transfer co-design methods to a web-based setting. However, transforming all the co-design methods and techniques into a web-based setting may neither be possible nor necessary.

## Abbreviations

D-DEC: Designing Discharge After Emergency Care
ED: emergency department
